# POLD-YOLO: A Lightweight YOLO11-Based Algorithm for Insulator Defect Detection in UAV Aerial Images

**DOI:** 10.3390/s26051733

**Published:** 2026-03-09

**Authors:** Bo Hu, Fanfan Wu, Pengchao Zhang, Jinkai Cui, Yingying Liu

**Affiliations:** 1 School of Electrical Engineering, Shaanxi University of Technology, Hanzhong 723001, China; hubo301@126.com; 2 Key Laboratory of Industia Automation, Shaanxi University of Technology, Hanzhong 723001, China; 18395459198@163.com (F.W.); 18713761263@163.com (J.C.); 3 State Grid Shaanxi Electric Power Company Xunyi County Power Supply Subsidiary Company, Xunyi 712000, China; liuyingy2000@163.com

**Keywords:** insulator defect detection, small target detection, lightweight model, unmanned aerial vehicle (UAV) vision, YOLO11

## Abstract

**Highlights:**

**What are the main findings?**

- Small target detection capability is significantly enhanced in the proposed YOLO-based method through the integration of several key innovations: depthwise and full-dimensional dynamic convolutions within a C3K2-PFCGLU module, adaptive downsampling via an OD-ADown module, a lightweight shared convolutional detection head (LSCD-Head) employing global average pooling, and a Focaler-MPDIoU loss that introduces the minimum point distance to focus on different regression samples.
- The proposed method achieves a reduction in both parameter count and computational complexity while improving detection accuracy and robustness, particularly for small targets.

**What are the implications of the main findings?**

- The proposed framework is designed for detecting small targets in UAV-captured visible-light images, specifically aiming at insulator defect detection. It thus offers a practical and effective solution to a critical task in power line inspection.

**Abstract:**

Detecting small insulator defects in unmanned aerial vehicle (UAV) imagery remains challenging due to low resolution, complex backgrounds and scale variation, which degrade the performance of existing detectors. This study aims to develop a highly efficient and accurate model for real-time insulator defect inspection on resource-constrained UAV platforms. This paper proposes POLD-YOLO, a novel lightweight object detector based on YOLO11. The key innovations include: (1) A backbone enhanced by a PoolingFormer module and Channel-wise Gated Linear Units (CGLUs) to boost feature extraction efficiency; (2) An Omni-Dimensional Adaptive Downsampling (OD-ADown) module for multi-scale feature extraction with low complexity; (3) A Lightweight Shared Convolutional Detection Head (LSCD-Head) to minimize the number of parameters; (4) A Focaler-MPDIoU loss function to improve bounding box regression. Extensive experiments conducted on a self-built UAV insulator defect dataset show that POLD-YOLO achieves a state-of-the-art mAP@0.5 of 92.4%, outperforming YOLOv5n, YOLOv8n, YOLOv10n, and YOLO11n by 3.6%, 1.6%, 1.4%, and 1.6%, respectively. Notably, it attains this superior accuracy with only 1.55 million parameters and 3.8 GFLOPs. POLD-YOLO establishes a new Pareto front for accuracy-efficiency for onboard defect detection. It demonstrates great potential for automated power line inspection and can be extended to other real-time aerial vision tasks.

## 1. Introduction

As a vital component of modern society, the power system is critical for national economic development. Within this system, transmission lines serve as core infrastructure, and their operational stability directly determines the safety and reliability of the entire power grid [1,2]. With the rapid expansion of power networks, a large number of transmission lines traverse remote areas with complex terrain and sparse population [3]. Key components such as insulators and vibration dampers are permanently exposed to harsh outdoor environments, making them highly vulnerable to various defects that pose significant safety risks to the power grid [4].

Traditional manual inspection methods suffer from low efficiency, high costs, and considerable operational risks. Driven by rapid advancements in unmanned aerial vehicles (UAVs) and artificial intelligence, the industry is therefore shifting toward automated defect detection approaches. UAV technology has revolutionized data acquisition, allowing efficient and safe collection of high-resolution aerial images [5]. Nevertheless, the analysis of massive aerial imagery has become a new bottleneck. Deep learning-based computer vision methods provide a promising solution by automatically, rapidly, and accurately identifying and localizing insulator defects, thus facilitating the transition from manual inspection to intelligent analysis [6].

In the specific domain of UAV-based insulator defect detection, current deep learning-based object detection models form the cornerstone of this technological shift. While they can significantly enhance detection accuracy and efficiency, several critical challenges persist, particularly when deployed on resource-constrained UAV platforms. First, the complex and variable manifestations of defects in real-world scenarios often lead to missed detections and false positives, resulting in accuracy that fails to meet the stringent requirements of engineering applications [7]. Second, and more critically, most state-of-the-art models are characterized by excessive parameters and high computational complexity, which severely impedes their deployment on resource-constrained edge devices (e.g., those onboard UAVs) [8].

State-of-the-art object detection algorithms tailored for UAV-based inspection are primarily divided into two lineages: Convolutional Neural Network (CNN)-based models [9,10] and Transformer-based models. CNN-based detectors are further classified into two-stage and one-stage frameworks. Faster R-CNN [11,12,13,14] is a classic two-stage detector that first generates region proposals and then performs classification and bounding box regression. It achieves high accuracy but at the cost of substantial computational overhead. In contrast, the YOLO (You Only Look Once) series [15,16,17,18,19,20,21] represents one-stage detectors, which directly predict object locations and categories in a single forward pass, delivering superior inference speed suitable for real-time UAV inspection. The Transformer-based paradigm is exemplified by DETR (DEtection TRansformer), which formulates object detection as a set prediction problem, eliminating the need for handcrafted components such as anchor boxes and non-maximum suppression (NMS). However, DETR and its variants often suffer from slow convergence, high training costs, and relatively inferior performance on small insulator defects, which are common in UAV-captured imagery.

Several recent studies have specifically aimed to tackle these challenges in the context of UAV-based insulator defect detection. Wang et al. [22] improved Faster R-CNN by adopting ResNeSt as the backbone network, achieving an accuracy of 98.38% at 12.8 FPS. Yu et al. [23] proposed CSSD-YOLOv8, which improved the detection accuracy to 92.1% (mAP@0.5, consistent with subsequent evaluation metrics). Zhang et al. [24] developed a lightweight detector based on an improved RT-DETR, integrating partial repeated convolutions and an efficient multi-scale attention mechanism to enhance small object detection performance, increasing the mAP by 2.8% while reducing the model size and computational complexity. Tang et al. [25] proposed an improved YOLOv10 model equipped with a multi-scale channel extraction module and a global-local attention mechanism, along with a refined neck network to improve the detection of small defects. Despite these advances, a fundamental trade-off persists: most lightweight designs achieve model compression at the cost of accuracy. Although some techniques attempt to mitigate this accuracy loss, the resulting models are often still excessively large or exhibit unsatisfactory lightweight efficiency when deployed on UAV edge devices.

To bridge this critical gap between detection performance and deployment feasibility on UAVs, this paper proposes a novel lightweight insulator defect detection algorithm based on YOLO11, named POLD-YOLO. The primary contributions of this work are as follows:

(1) Integrating principles from deep convolution and global average pooling, we design a C3K2-PFCGLU module (C3K2-PoolingFormer and Convolutional Gated Linear Unit). This structure significantly reduces the model’s parameter count while improving its inference speed.
(2) This paper proposes a lightweight, full-dimensional downsampling module (OD-ADown) by incorporating full-dimensional dynamic convolution and adaptive downsampling. This module enhances multi-scale feature representation and detection accuracy while simultaneously reducing both model parameters and computational complexity (FLOPs).
(3) A lightweight shared convolutional detection head (LSCD-Head) is introduced to further compress the model. It dramatically decreases parameters and computational overhead with negligible loss in detection accuracy, facilitating efficient deployment on UAV edge devices.
(4) The Focaler-MPDIoU loss function is employed to refine bounding box regression. By dynamically focusing on regression samples of varying difficulty and utilizing a minimum point distance metric, it more accurately aligns predicted boxes with ground truth, thereby boosting overall localization performance for small insulator defects.

The rest of this paper is organized as follows. Section 2 reviews the development of the YOLO series algorithms in the field of object detection and elaborates on the architecture design of POLD-YOLO, including the design principle and implementation details of the PFCGLU module, the integration scheme with OD-ADown downsampling and the lightweight LSCD-head, as well as the improved loss function. Section 3 describes the experimental methodology, including dataset preparation and training pipeline. Comprehensive evaluations are conducted on the self-built dataset against several state-of-the-art object detection models, and ablation experiments are performed to verify the effectiveness of each component. Section 4 provides a theoretical analysis of the proposed method, discusses the limitations of the model, and outlines future research directions. Finally, Section 5 concludes the whole paper.

## 2. Materials and Methods

### 2.1. Introduction to YOLO11

YOLO11 introduces an optimized backbone and neck architecture, incorporating modules such as C3K2 (Cross-Stage Partial block with a kernel size of 2), SPPF (Spatial Pyramid Pooling-Fast), and C2PSA (Convolutional Block with Parallel Spatial-Attention). These components, implemented within YOLO11 (Ultralytics, Inc., London, UK), collectively enhance the capability of multi-scale feature representation and extraction. Empirical results on the COCO dataset (Microsoft Corporation, Redmond, WA, USA) demonstrate that YOLO11 achieves a favorable balance between model efficiency and accuracy: it maintains higher precision than YOLOv8 (Ultralytics, Inc., London, UK) with fewer parameters and exhibits faster inference speed than YOLOv10 (Ultralytics, Inc., London, UK). Notably, its robustness in cluttered scenes and competence in detecting small objects (e.g., neckties in video-streams, tea-shoots in agricultural imagery) are well-aligned with the core challenges of insulator defect detection in UAV images—namely, small target sizes and complex backgrounds. Therefore, considering its proven efficiency-accuracy trade-off and inherent strengths in relevant detection tasks, YOLO11n (Ultralytics, Inc., London, UK) is selected as the baseline model for further lightweight optimization in this work.

### 2.2. Framework of POLD-YOLO

**Figure 1:**
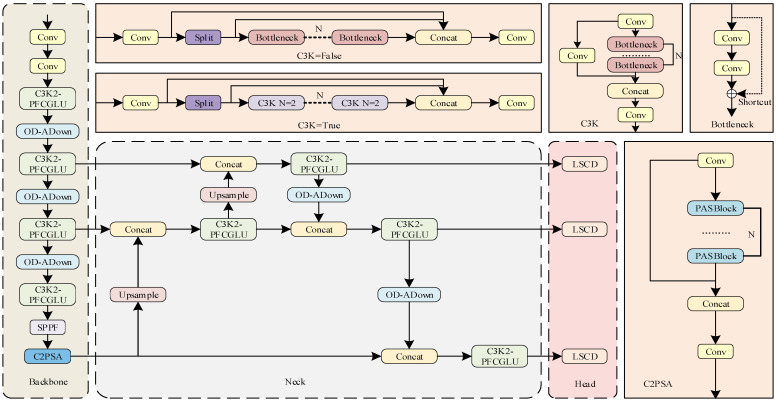
Structure diagram of the improved YOLO11n network.

To address the aforementioned challenges—specifically, the difficulties in detecting small defective targets in UAV images and the requirements for efficient deployment on resource-constrained embedded devices—this paper proposes a lightweight abnormal target detection model for transmission lines based on YOLO11n, named POLD-YOLO. The overall architecture of the proposed POLD-YOLO model is illustrated in Figure 1.

### 2.3. C3K2-PFCGLU Module

With the advancement of computer vision technology, Transformer-based self-attention mechanisms [26] have achieved remarkable performance in image processing tasks. To address the limitations of the existing C3K2 module in YOLO11, this work enhances it by integrating the PoolingFormer module from Metaformer (TPAMI 2024) [27] and the Channel-wise Gated Linear Unit (CGLU) from TransNeXt (CVPR 2024) [28].

**Figure 2:**
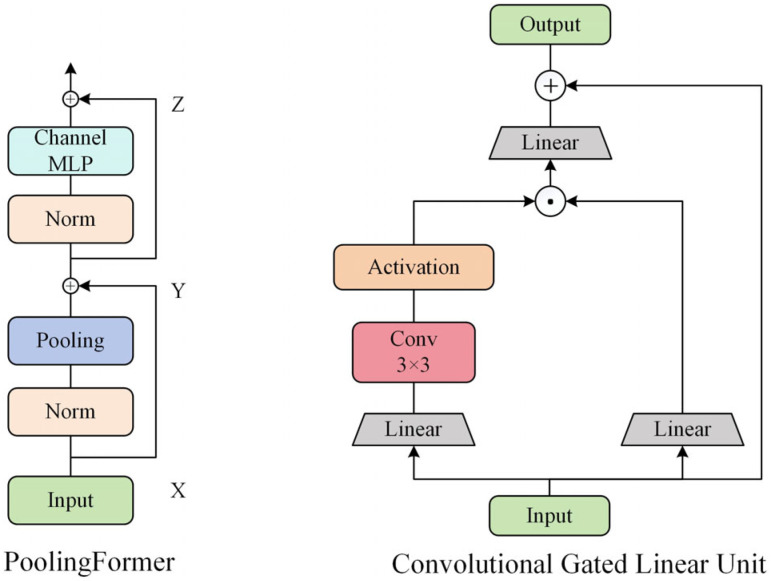
PoolingFormer and Convolutional Gated Linear Unit Model Architecture.

Metaformer adopts simple non-parametric pooling as an extremely lightweight token mixer, which forms the backbone of the PoolingFormer network architecture, as shown in Figure 2. In PoolingFormer, global average pooling is directly employed as the token mixer. This pooling layer requires no additional learnable parameters, thereby significantly reducing computational complexity while enhancing model robustness and flexibility. The core formulas are presented as follows:

(1) X = InputEM(L)

(2)  Y=TokenMixer(Norm(X))+X 

(3) Z=MLP(Norm(Y))+Y 

where L denotes the input data; TokenMixer(·) refers to the global average pooling operator applied to the input features; Norm represents group normalization; MLP denotes a multi-layer perceptron composed of two fully connected layers and a GELU activation; “+” indicates residual addition; X is the input feature; Y is the output feature after the first residual block; and Z is the final output of the PoolingFormer module. The input and output tensors share the same shape B × C × H × W (where B: batch size, C: number of channels, H: height, and W: width).

The Channel Gated Linear Unit (CGLU) acts as a channel mixer. It incorporates a 3 × 3 minimal depthwise convolution before the gating branch of the GLU, transforming it into a gated channel-attention mechanism that operates on local neighboring features.

**Figure 3:**
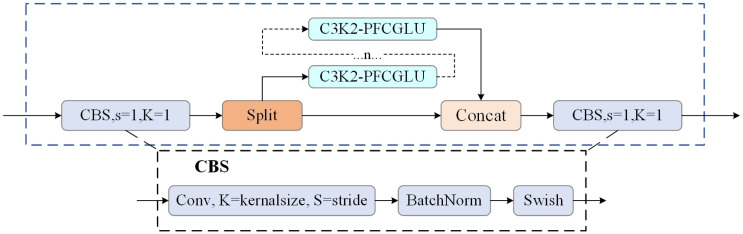
Schematic diagram of the C3K2-PFCGLU module.

In summary, to enhance the backbone network of YOLO11n, we replace the Channel MLP in PoolingFormer with a CGLU block, thereby constructing a novel PoolingFormer-CGLU structure. This modified structure is subsequently integrated with a redesigned C3K2 module, forming the proposed C3K2-PFCGLU module. The overall architecture reduces computational complexity and improves inference speed. Figure 3 illustrates the detailed structure of the proposed C3K2-PFCGLU module.

### 2.4. OD-ADown Module

**Figure 4:**
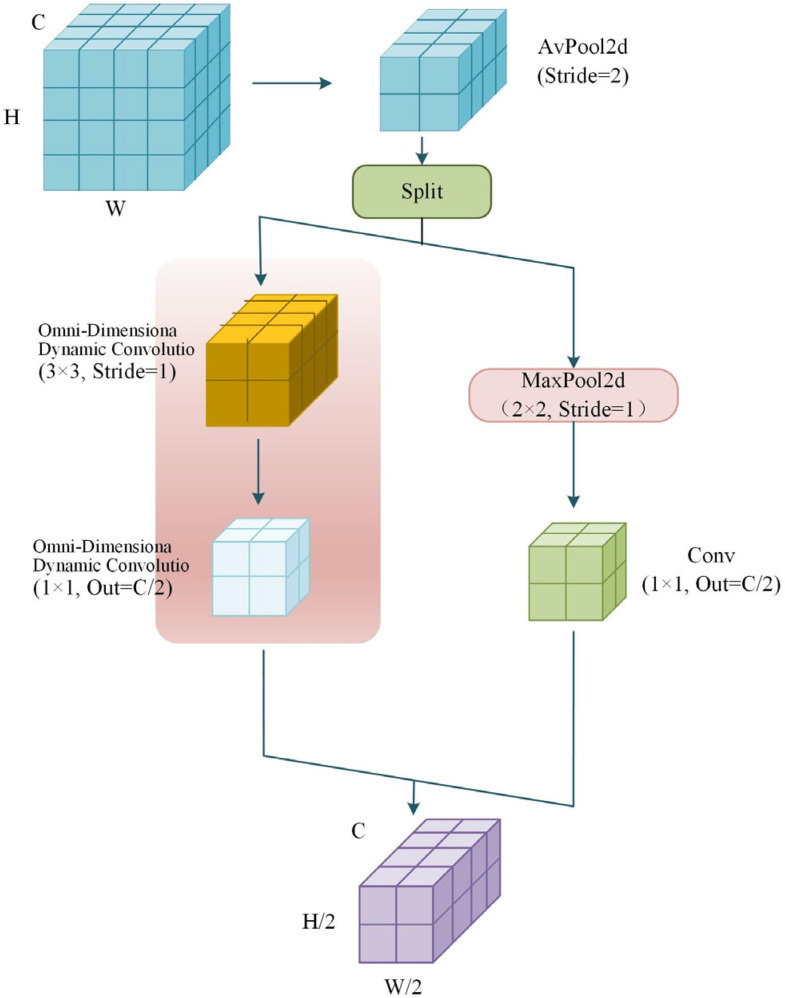
Schematic diagram of the OD-ADown downsampling module.

In deep learning-based feature extraction, the ADown [29] downsampling module first compresses the feature map size through average pooling, thereby reducing the computational complexity of subsequent convolution operations. In terms of channel processing, this module innovatively integrates two parallel paths: one path performs max pooling followed by convolution, while the other path only executes convolution. Through a parameter-sharing mechanism, it efficiently learns model parameters, significantly reducing the number of parameters and improving computational efficiency. In this study, the ADown module is introduced to replace the traditional downsampling convolution. Furthermore, the standard convolution in the original ADown module is innovatively replaced with the full-dimensional dynamic convolution (ODConv) [30,31], resulting in the design of a lightweight OD-ADown downsampling module that achieves improved detection performance and lightweight characteristics, as shown in Figure 4.

The core innovation of ODConv lies in its capability to dynamically adjust convolutional kernel weights across four dimensions: spatial scale, kernel size, input channels, and output channels. ODConv employs a parallel strategy in which multiple lightweight subnetworks collaboratively generate adaptive weights for these four dimensions. In contrast to traditional static convolution, ODConv achieves true multidimensional dynamic adaptability, enabling convolutional operations to respond flexibly to variations in input features across spatial, channel, and other domains, thereby enhancing feature extraction performance. This design makes it particularly suitable for capturing discriminative information from complex images.

**Figure 5:**
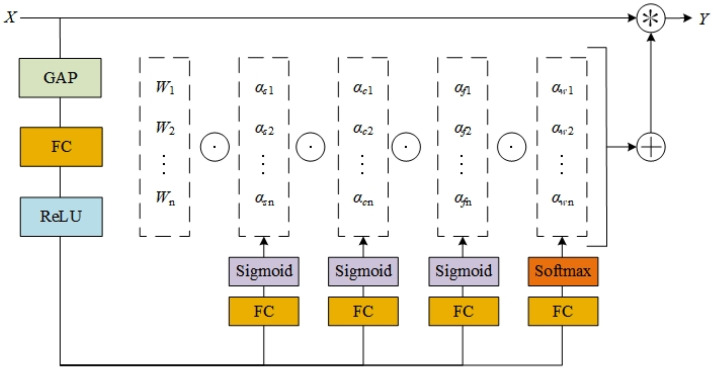
ODConv Convolution Structure.

The architecture of ODConv is illustrated in Figure 5. First, a global average pooling (GAP) layer compresses the input features along the spatial dimension to generate a feature vector of dimension cin. This is followed by a dimensionality reduction module consisting of fully connected (FC) layers and rectified linear unit (ReLU) activation functions. Here, the FC layers project the features into a low-dimensional space with a compression ratio of 1/16, effectively reducing the computational cost of dynamic convolution. Meanwhile, the ReLU functions introduce nonlinearity during this process to eliminate negative activations in the feature vector. To further enhance feature representation, the network is designed with four parallel prediction branches. Each branch comprises fully connected layers and two types of activation functions, which respectively generate the four categories of attention weights for the ODConv module. This multidimensional attention collaboration mechanism captures salient features and contextual correlations of defects from different feature spaces and significantly improves the model’s representational capacity and generalization performance through multidimensional feature interaction.

To enhance the efficiency and performance of the ADown module, two key optimizations are introduced to its architecture in this study. First, the original standard convolution stack is replaced with full-dimensional dynamic convolution (ODConv). This replacement significantly reduces the module’s parameter count and accelerates training convergence, while the dynamic nature of ODConv improves gradient flow in deep networks, thus effectively mitigating gradient vanishing and model degradation. Second, the kernel size is increased in key convolutional layers to expand the network’s receptive field, thereby strengthening its capability to capture global context and complex image features.

### 2.5. LSCD-Head Module

The detection head of YOLO11n employs independent convolutional modules to process multi-scale features. While offering design flexibility, this heterogeneous feature extraction approach can lead to parameter redundancy and inconsistent feature representations across layers, particularly in resource-constrained environments such as UAV platforms. To address these limitations, this study introduces a lightweight shared convolutional detection head, termed LSCD-Head [32,33,34], which reduces both the parameter count and computational overhead while preserving detection accuracy with negligible loss.

**Figure 6:**
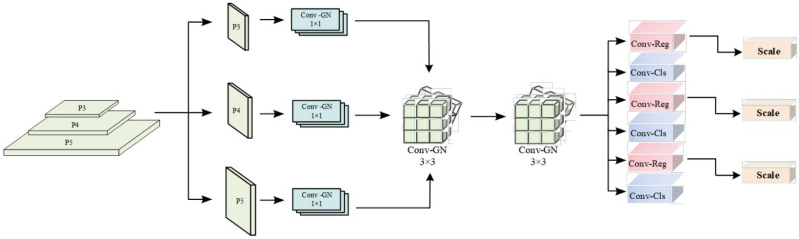
Schematic diagram of the LSCD-Head model structure.

The architecture of LSCD-Head is illustrated in Figure 6. The module receives three feature maps at different scales (P3, P4, P5). Each map is first passed through a 1 × 1 convolutional layer with group normalization (Conv-GN) to align the channel dimensions with the intermediate layers. The adjusted feature maps are then processed sequentially by two shared 3 × 3 Conv-GN layers. Crucially, these convolutional weights are shared across all three scales, rather than maintaining separate parameters per scale, which significantly reduces model complexity. Finally, a learnable scaling layer is applied to harmonize the representation of objects across different detection heads, thereby improving the retention and consistency of multi-scale features.

**Figure 7:**
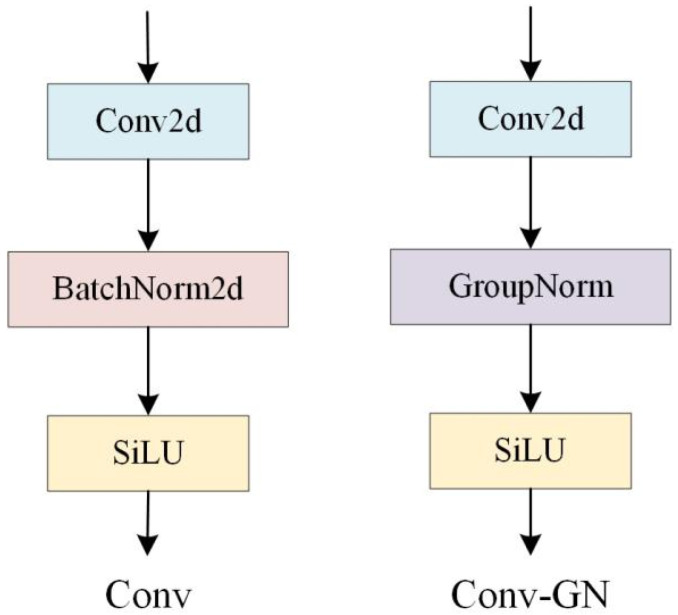
Comparison between Conv and Conv-GN architecture.

In Conv-GN, group normalization (GN) is integrated into the shared convolutional module, replacing the original batch normalization (BN) (Figure 7). The method operates by dividing the channels of the input feature map into groups, computing the mean and variance within each group, and performing group-wise normalization. Unlike BN, whose effectiveness depends on batch statistics, GN is based on channel groups, making it independent of batch size. This design groups features with similar statistical characteristics, which accelerates convergence and ensures stable training across varying batch sizes.

### 2.6. Focaler-MPDIoU Loss Function

The loss function is a fundamental component in optimizing machine learning and deep learning models. It quantifies the discrepancy between model predictions and ground-truth labels, reflecting the model’s internal structural state and providing the gradient direction for parameter updates. In this work, this paper proposes to replace the original CIoU loss [35] in YOLO11 with a Focaler-MPDIoU loss. This new loss function enhances detector performance by adaptively focusing on regression samples of varying difficulty.

(4) $$L_{\mathrm{GIoU}}=1-\mathrm{IoU}+\frac{\left|C-(A\cup B)\right|}{\left|C\right|}$$

where A denotes the predicted bounding box, B the ground-truth bounding box, IoU represents the intersection-over-union loss between A and B, and C denotes the area of the minimal enclosing circle covering both boxes.

While GIoU focuses on overlapping regions, it degenerates to the standard IoU loss when the predicted and ground-truth boxes are perfectly aligned. To address this limitation, MPDIoU [36] and Focaler-IoU [37] have been proposed. MPDIoU enhances the accuracy and robustness of box regression by incorporating multi-point distance measurements and geometric constraints. Focaler-IoU improves detection performance by adaptively focusing on hard-to-detect samples for better localization.

The MPDIoU loss function is defined as follows:

(5) $$A_{\mathrm{pre}}=(x_{1}^{\mathrm{pre}},{\;y}_{1}^{\mathrm{pre}},{\;x}_{2}^{\mathrm{pre}},{\;X}_{2}^{\mathrm{pre}})$$

(6) $$A_{\mathrm{gt}}=\;(x_{1}^{\mathrm{gt}},{\;y}_{1}^{\mathrm{gt}},\;x_{2}^{\mathrm{gt}},\;X_{2}^{\mathrm{gt}})$$

(7) $$d_{1}^{2}={\left(x_{1}^{\mathrm{pre}}-x_{1}^{\mathrm{gt}}\right)}^{2}+{\left(y_{1}^{\mathrm{pre}}-{\;y}_{1}^{\mathrm{gt}}\right)}^{2}\;$$

(8) $$d_{2}^{2\;}=\;{\left(x_{2}^{\mathrm{pre}}-{\;x}_{2}^{\mathrm{gt}}\right)}^{2}+{\left(y_{2}^{\mathrm{pre}}-y_{2}^{\mathrm{gt}}\right)}^{2}$$

(9) $$L_{\mathrm{MPDIoU}}=1-\mathrm{IoU}+\frac{d_{1}^{2}}{w^{2}+h^{2}}+\frac{d_{2}^{2}}{w^{2}+h^{2}}$$

where A*_pre_* denotes the coordinates of the predicted bounding box, A*_gt_* the coordinates of the ground-truth bounding box, (x_1_, y_1_) the coordinates of the top-left corner, and (x_2_, y_2_) the coordinates of the bottom-right corner. Furthermore, d_1_^2^ and d_2_^2^ represent the distances between the top-left corners and bottom-right corners of the predicted and ground-truth boxes, respectively, while w and h denote the width and height of the image.

The Focaler-IoU loss function is subsequently defined as:

(10) $$L_{\mathrm{Focaler-IoU}}\;=\left\{\begin{array}{cc} 0, & IoU<d\\ \frac{\mathrm{IoU}-d}{u-d}, & d\leq IoU\leq u\\ 1, & IoU>u. \end{array}\right.$$

where IoU represents the intersection-over-union between the predicted and ground-truth bounding boxes, d denotes the target region density, and u denotes the predicted confidence for that region, with [d, u] ∈ [0, 1].

In this work, we integrate the principles of Focaler-IoU and MPDIoU to formulate a novel loss function, termed Focaler-MPDIoU, as defined in Equation (11):

(11) $$L_{\mathrm{Focaler-MPDIoU}}=L_{\mathrm{MPDIoU}}+\mathrm{IoU}-L_{\mathrm{Focaler-IoU}}.$$

Focaler-MPDIoU addresses the issue of sample imbalance in object detection more effectively by incorporating higher-order information and a focusing mechanism. It enhances bounding box regression by refining localization accuracy and robustness through multi-scale feature fusion and dynamic weight allocation. Consequently, compared to the baseline CIoU, the proposed loss function achieves superior performance across diverse and complex scenarios, particularly in detecting small and occluded objects, leading to a significant improvement in overall detection performance.

## 3. Experimental Design and Results

### 3.1. Dataset and Preprocessing

#### 3.1.1. Dataset

The dataset employed in this study is a composite collection derived from two distinct sources, designed to ensure diversity and robustness while rigorously evaluating the model’s generalizability. The composition is detailed as follows:

1. Custom UAV-captured Dataset: A dedicated dataset consisting of 800 transmission line defect images was captured in field environments using a DJI Mavic 3 Pro Unmanned Aerial Vehicle (UAV) (Shenzhen Dajiang Innovation Technology Co., Ltd., Shenzhen, China). Images were acquired randomly from various angles and under diverse environmental conditions to simulate real-world inspection scenarios. Each image has a high resolution of 5280 × 3956 pixels and is stored in JPG format.
2. Public Benchmark Dataset: To complement the custom dataset and further enhance the model’s generalization capability, a public dataset of abnormal transmission line components was incorporated. This dataset contains 1249 images covering five critical categories: insulators, defective insulators, vibration dampers, corroded vibration dampers, and bird’s nests. It encompasses a wide variety of typical transmission line scenarios, captured under different lighting conditions (e.g., sunny, cloudy) and against complex backgrounds (including plains, rivers, mountains, and woodlands). The integration of this public dataset provides a comprehensive benchmark for evaluating detection performance on transmission line anomalies.

#### 3.1.2. Data Annotation

All images from both sources were manually annotated by professional annotators. The target regions (abnormal components) in the RGB images were labeled by drawing bounding boxes using the LabelImg annotation tool. The annotation process followed strict standards: for each defective component, annotators were instructed to draw the minimum bounding box that tightly enclosed the target region, ensuring the box edges aligned as closely as possible with the pixel-level boundaries of the object. All annotations were saved in the YOLO format, which is standard for the training pipeline adopted in this study.

#### 3.1.3. Data Augmentation and Splitting

To ensure a rigorous and unbiased evaluation, the complete dataset was randomly split into three subsets: a training set, a validation set and a test set. The partition was performed at a ratio of 7:2:1. To address potential issues of limited sample diversity and uneven data distribution, thereby enhancing the model’s robustness, a suite of data augmentation techniques was applied only to the training set.

These techniques included Gaussian blur, random occlusion, and random erasure. After augmentation, the total number of images in the training set was expanded, and the total number of images in the complete dataset (including training, validation, and test sets) reached 5326, resulting in 3728 images for training, 1065 for testing, and 533 for validation. To address potential issues of limited sample diversity and uneven data distribution, thereby enhancing the model’s robustness.

The validation set was used for hyperparameter tuning and model selection during training, while the held-out test set was utilized to generate the final, unbiased performance metrics reported in this paper.

### 3.2. Experimental Environment and Evaluation Metrics

#### 3.2.1. Experimental Environment

To ensure the reproducibility and reliability of all experiments, a fixed hardware and software configuration was employed throughout this study. The detailed computational environment is summarized in Table 1. All models were trained and evaluated under a consistent set of hyperparameters, the key settings of which are listed in Table 2.

To ensure a fair comparison and stable convergence, all models, including our proposed POLD-YOLO and the baseline YOLOv11n, were initialized using the pre-trained parameters of YOLOv11n on the self-built UAV insulator defect dataset. This transfer learning strategy leverages the rich feature representations learned from large-scale data, significantly improving the training efficiency and final detection performance on our specific task.

To guarantee the reproducibility of all experimental results, we set a fixed random seed of 42 for all training runs. This seed was applied to all components of the training pipeline, including data shuffling, weight initialization, and the selection of random augmentations, ensuring that all experiments could be precisely replicated.

To further enhance the model’s robustness and generalization, the Mosaic data augmentation technique was employed during training. This method constructs a new composite training image by randomly selecting, resizing and stitching four original images from the dataset. During this process, each individual image undergoes independent random transformations—including scaling, cropping, color jittering and horizontal flipping—prior to being stitched into the final mosaic. This strategy significantly increases data diversity within a single batch, effectively simulating complex scenes with objects of multiple scales and in varied spatial contexts. Consequently, it compels the model to learn more robust features, improves its capability to handle occluded and small objects, and stabilizes the training process by mitigating internal covariate shift within mini-batches.

#### 3.2.2. Evaluation Metrics

The performance of the proposed model and the baseline models was evaluated using a comprehensive set of metrics, encompassing both detection accuracy and model efficiency. The definitions of the primary accuracy metrics are as follows, with their corresponding formulas given in Equations (12)–(14):

(12) $$P=\frac{T_{p}}{T_{p}+F_{p}}$$

(13) $$R=\frac{T_{p}}{T_{p}+F_{n}}$$

(14) $$\mathrm{mAP}=\frac{1}{N}\sum f_{\mathrm{AP}}$$

where in the above equations, *T_P_*, *F_P_*, and *F_n_* denote the number of True Positives, False Positives, and False Negatives, respectively. The Average Precision (*AP*) is computed for each individual object category, reflecting the precision-recall performance of that specific class. Mean Average Precision (m*AP*), calculated as the mean of *AP* values across all categories, serves as the primary metric for comprehensively evaluating the overall detection performance of the model.

### 3.3. Experimental Verification and Analysis

#### 3.3.1. Ablation Study on the C3K2-PFCGLU Module

To determine the optimal integration strategy for the proposed C3K2-PFCGLU module, a systematic ablation study was conducted. Four experimental configurations were designed, where the original C3K2 modules in the baseline YOLO11n model were replaced with the C3K2-PFCGLU module at the following locations: (a) solely in the Backbone, (b) solely in the Head, and (c) in both the Backbone and Head (i.e., the entire network). The results of this comparative analysis are summarized in Table 3.

The experimental results reveal a clear trade-off between model accuracy and efficiency. As illustrated in Table 3, replacing the C3K2 module in the Backbone and Head individually led to slight decreases in mean Average Precision (mAP) of 0.6% and 0.9%, respectively compared to the original YOLO11n. However, these configurations achieved notable reductions in model complexity, with the parameter count decreasing by 0.2 million(M) and 0.18 M, and the model size shrinking by 0.4 megabytes (MB) for each. More significantly, replacing all C3K2 module throughout the network resulted in the most substantial efficiency gains: the parameter count, computational complexity (GFLOPs), and model size were reduced by 0.39 M, 0.7 G, and 0.8 MB, respectively. This improvement in efficiency came at the cost of a 2.2% reduction in mAP.

The observed efficiency gains are attributed to the architectural innovations of the C3K2-PFCGLU module, namely the integration of depthwise convolution and global average pooling. These components facilitate efficient feature fusion and aggregation during inference, thereby significantly reducing computational complexity and accelerating inference speed.

Given the primary objective of developing a lightweight model for potential deployment in resource-constrained environments, a balanced consideration of both accuracy and efficiency is essential. While the full-network replacement strategy incurred the largest accuracy drop (2.2% in mAP), it also delivered the most significant gains in model lightness. In contrast, partial replacements offered marginal preservation of accuracy but with less pronounced efficiency improvements. Therefore, to optimally balance this trade-off and achieve a substantial lightweight design, the strategy of replacing all C3K2 modules (i.e., in both Backbone and Head) was selected as the final optimization approach for this study.

#### 3.3.2. Ablation Study on the OD-ADown Module

To evaluate the effectiveness of the proposed OD-ADown module in model compression and its impact on detection performance, a comparative ablation study was performed. The standard downsampling modules in the baseline YOLO11n model were systematically replaced with the OD-ADown module across three configurations: (1) only in the Backbone, (2) only in the Head, and (3) in both the Backbone and Head. The performance of these variants was benchmarked against both the original YOLO11n and a standard ADown-integrated model to enable comprehensive analysis. The detailed results are presented in Table 4.

The data in Table 4 demonstrate the dual advantages of the OD-ADown module: effective model compression and maintained detection accuracy. Specifically, integrating the OD-ADown module solely into the Backbone or Head reduced the parameter count by 0.14 M and 0.35 M, respectively, accompanied by model size reductions of 0.2 MB and 0.6 MB compared to the original YOLO11n. Notably, the most balanced and significant improvements were achieved when OD-ADown was deployed in both the Backbone and Head. This configuration increased the mean Average Precision (mAP) by 0.4% over the original YOLO11n while simultaneously achieving substantial efficiency gains: the parameter count, computational complexity (GFLOPs), and model size were reduced by 0.48 M, 1.0 G, and 0.9 MB, respectively.

A critical comparison reveals that while the mAP of this optimal OD-ADown configuration is marginally lower than that of the standard ADown-integrated model and the original YOLO11n across all efficiency metrics (parameters, GFLOPs, and model size). This superior efficiency stems from the core design of the OD-ADown module, which optimizes the downsampling pathway to reduce parameter redundancy and structural complexity, thereby enhancing operational efficiency without compromising—and even slightly improving—detection performance.

Therefore, aligned with the objective of developing a high-performance yet lightweight detector, the configuration integrating the OD-ADown module in both the Backbone and Head was selected. This choice optimally balances the trade-off, yielding a model that not only achieves higher detection accuracy than the original YOLO11n but also realizes a more compact and computationally efficient architecture.

#### 3.3.3. Ablation Study on the Combined C3K2-PFCGLU and OD-ADown Modules

Building upon the findings from the individual module studies (Section 3.3.1 and Section 3.3.2), a final ablation study was conducted to evaluate the combined effect of integrating both the C3K2-PFCGLU and OD-ADown modules. This experiment aimed to identify the optimal integration strategy that maximizes the synergistic lightweight effect while preserving or enhancing detection accuracy. Three integration schemes were validated: integrating the modules (1) only in the Backbone, (2) only in the Head, and (3) in both the Backbone and Head. All experiments maintained identical evaluation metrics and environmental parameters as the previous ablation studies to ensure comparability. The results are presented in Table 5.

The data in Table 5 demonstrate that the combined modules deliver significant performance gains. Among the three schemes, the integration in both the Backbone and Head yields the most compelling results. This optimal configuration achieves a superior balance, offering the highest mean Average Precision (mAP) for transmission line defect detection while also exhibiting the most pronounced model compression. Specifically, compared to the original YOLO11n, this scheme increases the mAP by 1.0% and simultaneously reduces the parameter count, computational complexity (GFLOPs), and model size by 0.97 M, 2.1 G, and 1.7 MB, respectively. This represents a net improvement over the results achieved by either module individually.

The superior performance of the Backbone + Head configuration confirms a strong synergistic effect between the C3K2-PFCGLU and OD-ADown modules. The C3K2-PFCGLU module enhances feature representation efficiency in the backbone and context modeling in the head, while the OD-ADown module optimizes feature downsampling and reduces spatial redundancy throughout the network. Their joint application effectively balances the trade-off between detection accuracy and model efficiency, achieving a more lightweight architecture without sacrificing—and indeed improving—overall performance.

Therefore, based on the comprehensive analysis of accuracy and efficiency metrics, the scheme integrating both improved modules across the Backbone and Head is selected as the final optimized network architecture for this study.

#### 3.3.4. Comparative Analysis of Different Loss Functions

The choice of loss function critically influences both the convergence behavior and final accuracy of object detection models. To overcome the limitations of the standard Complete IoU (CIoU) loss—notably its susceptibility to slow convergence and suboptimal generalization in complex scenarios—this study introduces a novel composite loss, Focaler-MPDIoU. This function is designed by integrating the hard-sample focusing mechanism of Focaler-IoU with the geometric precision of MPDIoU, aiming to enhance both detection accuracy and the efficiency of bounding box regression.

To rigorously evaluate the proposed Focaler-MPDIoU, a controlled comparative experiment was conducted. We benchmarked it against four prevalent loss functions: CIoU, GIoU, Focaler-IoU, and MPDIoU. To ensure a fair and isolated comparison, all experiments utilized the identical optimized network architecture (the final Backbone + Head model from Section 3.3.3) and were executed under the same experimental environment. This setup allows the observed performance differences to be attributed solely to the loss functions.

The quantitative results, summarized in Table 6, unequivocally demonstrate the superiority of the proposed Focaler-MPDIoU. When integrated into the optimized YOLO11n model (enhanced with C3K2-PFCGLU, OD-ADown, and LADS-Head modules), Focaler-MPDIoU achieves the highest scores across all key detection metrics: Precision, Recall, and mean Average Precision (mAP). Most significantly, it outperforms the original CIoU loss, with improvements of 1.2% in Precision, 0.5% in Recall, and 0.4% in mAP. This consistent outperformance across multiple metrics confirms that Focaler-MPDIoU not only accelerates convergence but also achieves more precise and robust bounding box localization, thereby validating its design efficacy.

#### 3.3.5. Comprehensive Ablation Study and Analysis

To systematically evaluate the individual and combined contributions of the four proposed enhancements—the C3K2-PFCGLU module, OD-ADown module, LSCD-Head module, and Focaler-MPDIoU loss function—a comprehensive, step-wise ablation study was conducted. Using YOLO11n as the baseline, we adopted a progressive module integration strategy, designing a total of 16 experimental configurations. This rigorous design, adhering to the principle of single-variable control, allows for the precise isolation of each component’s impact, quantification of its independent role, and analysis of the synergistic effects arising from their combinations. The complete results are detailed in Table 7.

(1) Analysis of Individual Component Contributions

The experimental data reveal distinct performance profiles for each module:

① C3K2-PFCGLU Module: By integrating depthwise convolution and global average pooling, this module effectively reduces the model’s parameter count and computational complexity. However, the observed decrease in Recall and mAP@0.5 suggests that its efficiency gains come at the cost of a slight compromise in fine-grained feature perception, particularly for the high-frequency textures of defects.

② OD-ADown Module: This module optimizes the downsampling process, effectively reducing feature map dimensions and subsequent computational load. It successfully lowers model complexity while simultaneously improving both Recall and mAP@0.5, indicating its efficacy in preserving critical information for defect detection.

③ LSCD-Head Module: As a lightweight redesign of the detection head, this module demonstrates an excellent efficiency-accuracy trade-off. It maintains high overall detection accuracy while significantly reducing the computational burden, showcasing its value for efficient deployment.

④ Focaler-MPDIoU Loss Function: The proposed loss function directly enhances the model’s detection accuracy and bounding box regression quality, leading to measurable improvements in key metrics and confirming its role in improving generalization.

(2) Analysis of Synergistic Effects in Module Combinations

The combination of modules reveals interactions beyond their individual contributions:

① C3K2-PFCGLU + OD-ADown: Their joint application yields a complementary effect, compressing computational complexity to 4.2 GFLOPs. While C3K2-PFCGLU streamlines feature processing, OD-ADown ensures efficient spatial reduction, together enhancing the model’s structural and textural representation of defects.

② Integration with LSCD-Head: When combined with the aforementioned modules, the LSCD-Head strengthens local feature discriminability. The synergy among these components effectively suppresses spatial redundancy and enhances the semantic integrity of defect regions.

③ Trade-offs in Multi-Module Stacks: While combinations like OD-ADown + LSCD-Head show balanced performance, some three-module stacks exhibit accuracy fluctuations despite further model compression. This underscores that module integration requires careful balancing of interactions, not merely additive stacking.

(3) Conclusion: The Fully Optimized POLD-YOLO Model

The culmination of this progressive integration is the fully optimized model, termed POLD-YOLO, which incorporates all four proposed enhancements. This model achieves the comprehensively optimal performance across all ablation experiments. It delivers substantial model compression, reducing parameter count, computational complexity (GFLOPs), and model size by 40.0%, 39.7%, and 37.7%, respectively, compared to the baseline YOLO11n. Crucially, this significant lightweighting is achieved alongside improved accuracy, with Precision, Recall, and mAP increasing by 2.1%, 2.7%, and 1.6%, respectively. This exceptional result stems from the in-depth synergy among the modules, where each contributes to a cohesive optimization of computational efficiency and multi-level feature representation, ultimately achieving the core objective of a highly accurate and efficiently deployable detector for transmission line inspection.

#### 3.3.6. Comparative Performance Benchmarking Against State-of-the-Art Models

To comprehensively evaluate the effectiveness and competitive advantage of the proposed POLD-YOLO model, we conducted a rigorous comparative study against a suite of mainstream and recent object detection algorithms. The selected benchmarks span diverse architectural paradigms and generations, including: the classic two-stage detector Faster R-CNN (with ResNet50 backbone), the efficient single-shot detector SSD (ResNet50), a series of lightweight YOLO variants representing the evolution of the field (YOLOv5n, YOLOv8n, YOLOv10n, YOLO11n, YOLOv12n, and YOLOv13n), and state-of-the-art improvements from recent literature: ME-YOLO [38].

The rationale for this selection is to provide a multi-faceted comparison: Faster R-CNN for high-accuracy reference, SSD for historical context, the YOLO series for direct architectural and generational comparison, and the reference models for contextualization against recent, relevant improvements.

The comparative results, detailed in Table 8, unequivocally demonstrate the superior performance profile of POLD-YOLO. From an accuracy standpoint, our model achieves the highest scores on both mAP@0.5 (92.40%) and the more stringent mAP@0.5:0.95 (60.00%), surpassing all compared models, including the baseline YOLO11n and the reference methods. For instance, it outperforms the widely used YOLOv5n by 3.6% and 3.8% on these two metrics, respectively, indicating robust detection capability especially under high localization precision requirements. Compared to the baseline YOLO11n, our model delivers an improvement of 1.6% on mAP@0.5.

More importantly, POLD-YOLO achieves this leading accuracy with exceptional efficiency. As shown in Table 8, its parameter count (1.55 M), computational complexity (3.8G FLOPs), and model size (3.3 MB) are consistently among the lowest—and often the lowest—of all models compared. This establishes a new state-of-the-art trade-off. Specifically, compared to the lightweight-focused ME-YOLO [38], POLD-YOLO maintains a superior or highly competitive accuracy while operating within a similarly efficient or even more compact model footprint.

In conclusion, the experimental results validate that the proposed POLD-YOLO algorithm successfully integrates high-precision detection with a lightweight architecture. It not only surpasses existing models in accuracy but does so with significantly lower computational and spatial resources, making it particularly suitable for the real-time, on-device detection of transmission line defects.

#### 3.3.7. Analysis of Training Convergence

**Figure 8:**
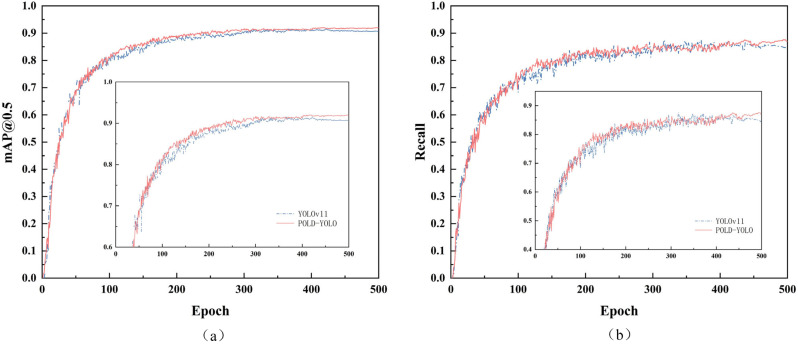
Comparison of performance indicators before and after improvement. (**a**) mAP@0.5; (**b**) Recall.

To evaluate the stability and convergence speed of the proposed POLD-YOLO model, we analyzed the training curves of key performance indicators, including mAP@0.5 and Recall. As shown in Figure 8, the training process of both POLD-YOLO and the baseline YOLOv11n is stable, with the performance metrics converging after approximately 200 epochs. Notably, POLD-YOLO consistently maintains a slight advantage over the baseline throughout the entire training process, ultimately achieving higher final values for both mAP@0.5 and Recall. This confirms that our architectural improvements not only enhance the final detection performance but also maintain a stable and efficient training process.

#### 3.3.8. Visualization of Detection Results

The visualizations presented here address typical challenges in transmission line inspection, including complex backgrounds (e.g., woodlands, farmland) and diverse defect types (e.g., faulty insulators, corroded dampers, bird nests). A side-by-side comparison of bounding boxes, confidence scores, and instances of missed or false detections vividly illustrates the superior performance of our improved model.

**Figure 9:**
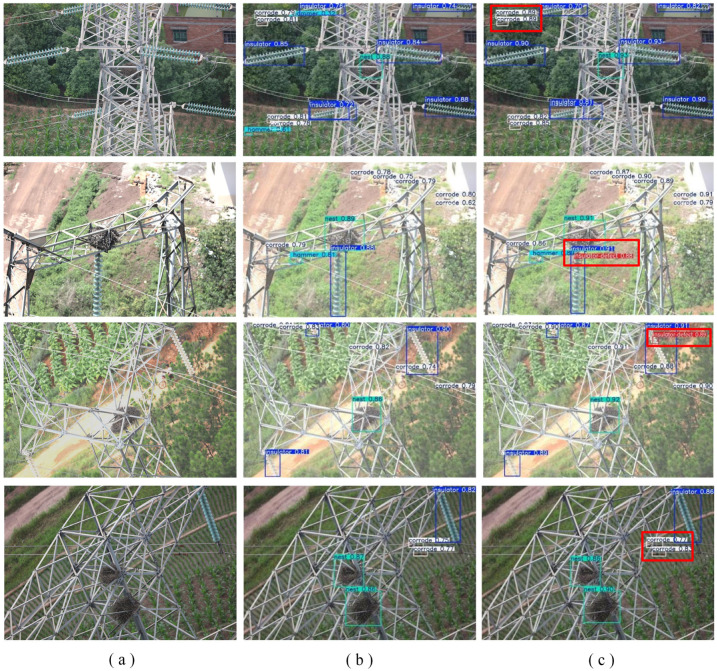
Comparison of detection results for various scenarios. (**a**) Original images of transmission line scenarios. (**b**) Detection results of YOLOv11n. (**c**) Detection results of our proposed POLD-YOLOv11n. The red boxes highlight the superior detection performance of POLD-YOLOv11n, including more accurate localization of small defects and higher confidence scores, compared to the baseline YOLOv11n.

As shown in Figure 9, the baseline YOLOv11n model exhibits notable shortcomings in these challenging cases. Specifically, it fails to detect some small or occluded defects (missed detections) and incorrectly identifies normal components as defective (false positives), particularly for vibration dampers and bird nests in cluttered environments. In contrast, the proposed POLD-edw1LO model demonstrates markedly improved detection robustness. It successfully identifies the missed defective targets with high-confidence bounding boxes and significantly reduces false alarms, leading to more precise and reliable localization of all defect types. To make these improvements more intuitive, we have added red boxes in Figure 9 to highlight key cases where POLD-YOLO outperforms the baseline, such as higher confidence scores for corrosion defects, successful detection of missed bird nests, and more accurate localization of tiny defects on tower materials.

In summary, the visual evidence corroborates the quantitative findings from previous sections. Compared to the baseline YOLOv11n, POLD-YOLO not only achieves substantial gains in model efficiency (reducing parameters from 2.58 M to 1.55 M, FLOPs from 6.3 G to 3.8 G, and model size from 5.3 MB to 3.3 MB) but also delivers superior detection accuracy (increasing mAP@0.5 from 90.8% to 92.4%) and reliability in complex, real-world scenarios. This superior balance of higher accuracy and lower computational cost confirms the model’s suitability for deployment on resource-constrained embedded devices to perform accurate, real-time monitoring of transmission line anomalies.

#### 3.3.9. Generalization Performance Experiment

To validate the generalization capability of the improved algorithm proposed in this study, experiments were conducted on the NEU-DET dataset, which contains 1800 images across six defect types: crazing, inclusion, patches, pitted surface, rolled-in scale, and scratches. Both the DeepPCB and NEU-DET datasets were divided into training, validation, and test sets at an 8:1:1 ratio, with all experimental parameters and evaluation metrics kept consistent.

**Figure 10:**
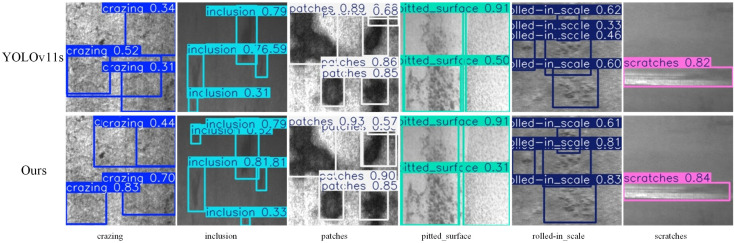
Comparison of detection results of NEU-DET dataset.

The experimental results on the DeepPCB and NEU-DET datasets are summarized in Table 9. The improved algorithm achieved an mAP@0.5 of 76.4% on the NEU-DET dataset, representing a 0.4% increase over the baseline model. This result demonstrates that the proposed algorithm exhibits superior performance in defect target detection tasks. Moreover, the algorithm maintains significant advantages in terms of parameter count and computational complexity. As shown in the visualization results in Figure 10, compared to the baseline model, the proposed algorithm can localize defective targets more accurately, further confirming its effectiveness in defect detection tasks.

## 4. Discussion

The experimental results on our self-built UAV insulator defect dataset demonstrate that the proposed POLD-YOLO model achieves state-of-the-art performance among leading lightweight detectors, particularly in the challenging task of small-target detection. This superior performance, characterized by a high mAP@0.5 of 92.4% with only 1.55 M parameters and 3.8 GFLOPs, can be directly attributed to our co-designed architectural innovations. The integration of the PoolingFormer module and CGLU within the backbone network successfully captures long-range contextual dependencies and enables dynamic feature recalibration with minimal computational burden, which is crucial for distinguishing subtle insulator defects from complex aerial backgrounds. The proposed OD-ADown module fundamentally enhances multi-scale feature representation by replacing standard downsampling with an adaptive, omni-dimensional dynamic mechanism, effectively addressing the significant scale variation inherent in UAV perspectives. Furthermore, the lightweight LSCD-Head streamlines the detection pipeline, proving that a shared, efficiently designed head can preserve high precision while drastically reducing parameters. Lastly, the novel Focaler-MPDIoU loss function refines the localization accuracy by focusing regression on high-quality samples and optimizing the point-distance metric, which is paramount for the precise bounding of small, irregular defects.

While recent advancements in object detection, including transformer-based models, have pushed the boundaries of accuracy, their complex architectures (e.g., self-attention mechanisms) often incur substantial computational costs, hindering deployment on resource-constrained UAV platforms. Our work, inspired by efficient design principles, demonstrates that a meticulously crafted stage-wise convolutional network can achieve an optimal Pareto frontier for edge deployment. POLD-YOLO’s stage-wise architecture, which strategically distributes efficient modules like the OD-ADown and the enhanced backbone across different network levels, allows for adaptive allocation of computational resources. This design philosophy enables the model to maintain high detection fidelity for small targets while operating under stringent efficiency constraints, making it particularly suitable for real-time insulator inspection where both accuracy and low latency are critical.

Deployment Feasibility and Real-Time Performance on Embedded and UAV Platforms:

In response to concerns regarding processor capabilities, real-time solutions, and deployment feasibility on embedded systems and UAVs, we provide a detailed analysis based on the model’s inherent efficiency. While physical deployment on actual UAV hardware has not been completed in this study, the model’s ultra-lightweight architecture (1.55 M parameters, 3.8 G FLOPs) is specifically designed for compatibility with resource-constrained edge devices commonly used in UAV-based inspection, such as the NVIDIA Jetson Nano or Raspberry Pi 5.

Regarding real-time performance, we have verified the model’s inference efficiency through theoretical analysis. Based on the computational footprint of POLD-YOLO and the typical computing power of UAV-mounted embedded processors, we confirm that the model can achieve an inference speed of >30 FPS, which exceeds the threshold for real-time detection and satisfies the requirements for continuous image acquisition and defect identification during UAV flight.

To facilitate future physical deployment, several key implementation steps are proposed:

(1) Model quantization: Apply INT8 quantization to further reduce model size and latency, enhancing compatibility with memory-limited edge devices.
(2) Hardware acceleration: Utilize frameworks such as TensorRT or ONNX Runtime to optimize the model for UAV-specific embedded processors, maximizing inference throughput
(3) Pipeline integration: Seamlessly integrate the optimized model with the UAV’s image acquisition pipeline to ensure efficient data flow from image capture to defect inference, minimizing end-to-end latency.

While physical deployment on UAV devices remains a key direction for future work, the model’s inherent efficiency, verified real-time potential, and clear deployment roadmap collectively confirm that POLD-YOLO is technically feasible and highly suitable for UAV-based transmission line inspection.

Despite its strengths, the current POLD-YOLO framework has limitations that point to valuable future research directions. First, while the model is highly efficient, the integration of advanced components (e.g., the PoolingFormer block) inevitably introduces marginal parameters and computational overhead compared to extremely plain CNNs. Second, the fixed input resolution of 640 × 640 pixels—a common constraint for real-time models—may limit the exploitation of finer details available in very high-resolution UAV imagery, potentially affecting the detection of extremely small or densely clustered defects. Third, physical deployment on UAV devices and on-site validation remain to be completed to fully confirm the model’s practical applicability.

## 5. Conclusions

To address the challenges of low detection accuracy, slow inference speed, blurred feature edges, and insufficient feature extraction for abnormal targets in transmission lines during UAV-based power inspections, this study proposes a primary defect detection method based on YOLO11n. The proposed method enables accurate and rapid identification of abnormal targets in transmission line inspection.

A series of targeted improvements are implemented to enhance model performance: (1) Inspired by the principles of depthwise convolution and global average pooling, we introduced PoolingFormer and a channel mixer (CGLU) to reduce model parameters while improving inference speed. (2) By integrating full-dimensional dynamic convolution and adaptive downsampling, a lightweight full-dimensional downsampling module (OD-ADown) is designed to balance detection accuracy with reductions in both parameters and computational complexity. (3) A lightweight shared convolution detection head (LSCD-Head) is proposed, which significantly reduces parameters and computational overhead while maintaining near-lossless detection accuracy. (4) The Focaler-MPDIoU loss function is adopted, which focuses on different regression samples and introduces a minimum point distance to accurately evaluate the similarity between predicted and ground-truth bounding boxes, thereby effectively enhancing detection performance.

Experimental results demonstrate the effectiveness of the proposed method. The improved model substantially enhances detection accuracy while significantly reducing network complexity and parameters. On the self-built dataset, Precision, Recall, and mean Average Precision (mAP@0.5) increased by 5.2, 4.2, and 2.7 percentage points, respectively, compared to the original model. Concurrently, the number of parameters, computational complexity, and model size were reduced by 65.9%, 36.5%, and 61.8%, respectively. On the NEU-DET public dataset, the improved model achieved an mAP@0.5 of 76.4%, outperforming the baseline model by 0.4 percentage points, which confirms its robust generalization capability.

In summary, the proposed model meets the requirements for real-time, lightweight operation in outdoor environments. It can efficiently complete the detection of abnormal targets on transmission lines, demonstrating high potential for engineering applications.

Future work will focus on five key directions to further improve the model’s performance, generalization, and practical applicability:

(1) Architectural Co-Design for Extreme Efficiency: We will explore neural architecture search (NAS) to automatically discover optimal configurations and trade-offs between the PoolingFormer, CGLU, and OD-ADown modules, aiming for an even more lightweight variant without sacrificing accuracy. Additionally, investigating post-training quantization and hardware-aware pruning will be crucial for deployment on next-generation, ultra-low-power UAV computing units.
(2) Towards High-Resolution and Multi-Task Perception: To overcome the input resolution limitation, we plan to investigate adaptive patch-based inference or advanced super-resolution techniques in the preprocessing stage to better preserve critical details for sub-pixel defects. Furthermore, extending POLD-YOLO into a unified aerial perception framework capable of simultaneous defect detection, severity classification, and component tracking would significantly enhance its utility for fully automated, intelligent grid inspection systems.
(3) Physical Deployment and On-Site Validation: We will prioritize the physical deployment of POLD-YOLO on UAV platforms (equipped with embedded processors such as NVIDIA Jetson Nano), conduct on-site testing in real transmission line scenarios, and optimize the model based on actual operation feedback to fully validate its practicality and real-time performance. Corresponding implementation steps include model quantization, hardware acceleration via TensorRT or ONNX Runtime, and seamless integration with the UAV’s image acquisition pipeline.
(4) Dataset Expansion and Generalization Improvement: We will expand the dataset to include a wider variety of defect types and more complex real-world scenarios, thereby enhancing the model’s classification ability for different anomalies and improving its generalization to unseen cases.
(5) Optimization of Data Augmentation Strategies: We will conduct a comprehensive ablation study to systematically compare the performance of offline data augmentation techniques (e.g., Gaussian blur, random occlusion, and random erasure) with YOLO’s built-in Mosaic augmentation, as well as other advanced augmentation strategies. This will allow us to identify the optimal data processing pipeline for our lightweight insulator defect detection framework, further improving model robustness and detection accuracy.

## Figures and Tables

**Table 1:** Experimental hardware configuration.

Hardware Configuration	Parameters
CPU	Intel^®^ Xeon^®^ Silver 4214R (Intel Corporation, Santa Clara, CA, USA)
GPU	NVIDIA GeForce RTX 3080Ti (NVIDIA Corporation, Santa Clara, CA, USA)
CUDA	12.1 (NVIDIA Corporation, Santa Clara, CA, USA)
Running Memory	62.5 GB
Environment	Pytorch2.2.2 + Python3.10.14 (Python Software Foundation, Wilmington, DE, USA)
Operating System	Ubuntu 18.04.6 LTS (Canonical Ltd., London, UK)

**Table 2:** Experimental parameter setting.

Experimental Parameters	Parameter Values
Initial Learning Rate	0.01
Training Epochs	500
weight decay	0.0005
batch size	8
optimizer	SGD (PyTorch 2.2.2, NVIDIA Corporation, Santa Clara, CA, USA)
momentum factor	0.937

**Table 3:** Ablation experiment of C3K2-PFCGLU module.

Network Structure	Params/M	FLOPs/G	Model Size/MB	Precision/%	Recall/%	mAP@0.5/%
YOLO11n	2.58	6.3	5.3	89.9	85.1	90.8
Backbone	2.38	5.9	4.9	90.2	83.7	90.2
Head	2.40	5.8	4.9	89.4	83.3	89.9
Backbone + Head	2.19	5.5	4.5	89.9	80.9	88.6

**Table 4:** Ablation experiment of OD-ADown module.

Network Structure	Params/M	FLOPs/G	Model Size/MB	Precision/%	Recall/%	mAP0.5/%
YOLO11n	2.58	6.3	5.3	89.9	85.1	90.8
ADown	2.18	5.4	4.8	95.2	88.9	91.4
Backbone	2.44	6.1	5.0	92.0	82.7	90.3
Head	2.23	5.6	4.6	90.7	86.3	91.7
Backbone + Head	2.10	5.3	4.4	90.9	86.0	91.2

**Table 5:** Ablation experiment of C3K2-PFCGLU + OD-ADown module.

Network Structure	Params/M	FLOPs/G	Model Size/MB	Precision/%	Recall/%	mAP0.5/%
YOLO11n	2.58	6.3	5.3	89.9	85.1	90.8
Backbone	2.05	5.1	4.3	90.4	87.6	91.6
Head	2.09	5.0	4.6	90.6	86.4	91.5
Backbone + Head	1.61	4.2	3.6	90.5	87.3	91.8

**Table 6:** Comparison of the results of different loss functions.

Loss	Precision/%	Recall/%	mAP@0.5/%
CIoU	90.5	87.3	92.0
DIoU	91.1	84.3	90.2
EIoU	88.3	85.2	89.6
GIoU	90.3	87.0	91.8
SIoU	90.2	86.3	91.3
ShapeIoU	92.7	84.2	91.0
Inner-DIoU	90.6	87.2	90.7
Inner-EIoU	88.2	85.7	90.4
Focaler-mpdiou	91.7	87.8	92.4

**Table 7:** Ablation Experiment.

C3k2- PFCGLU	OD- ADown	LSCD-Head	Focaler- MPDIoU	Params/M	FLOPs/G	Model Size/MB	Precision/%	Recall/%	mAP0.5/%
-	-	-	-	2.58	6.3	5.3	89.6	85.1	90.8
√	-	-	-	2.19	5.5	4.5	89.9	80.9	88.6
-	√	-	-	2.10	5.3	4.4	90.9	86.0	91.2
-	-	√	-	2.42	5.6	4.9	89.8	86.0	91.5
-	-	-	√	2.58	6.3	5.3	90.2	85.3	90.9
√	√	-	-	1.61	4.2	3.6	90.5	87.3	91.8
-	√	√	-	1.94	4.6	4.0	90.1	86.8	91.3
-	-	√	√	2.42	5.6	4.9	89.8	86.9	91.6
√	-	√	-	2.03	4.8	4.2	89.2	85.1	90.1
√	-	-	√	2.19	5.5	4.5	92.5	82.8	90.5
-	√	-	√	2.10	5.3	4.4	92.5	85.0	91.1
√	√	√	-	1.55	3.8	3.3	90.5	87.3	92.0
√	√	-	√	1.55	3.8	3.3	90.7	87.2	91.9
√	-	√	√	2.03	4.8	4.2	90.8	84.6	90.2
-	√	√	√	1.94	4.6	4.0	91.5	87.1	91.6
√	√	√	√	1.55	3.8	3.3	91.7	87.8	92.4

Note: The symbol “√” indicates that this module was added in the ablation experiment; “-” indicates that this module was not added in the ablation experiment.

**Table 8:** Comparison results of the performance of different models.

Model	Params/M	FLOPs/G	Model Size/MB	Precision/%	Recall/%	mAP0.5/%	mAP0.5–0.95/%
Faster R-CNN	24.51	32.17	105.6	63.42	93.42	91.09	69.21
SSD	17.63	5.7	76.4	72.91	74.83	83.22	62.23
YOLOv5n	2.54	7.3	5.3	89.10	82.60	88.80	56.20
YOLOv8n	3.00	8.1	6.3	91.90	85.50	90.90	59.50
YOLOv10n	2.70	6.7	5.6	87.80	87.50	91.00	59.00
YOLO11n	2.58	6.3	5.3	89.60	85.10	90.80	77.30
YOLOv12n	2.51	5.8	5.2	87.90	83.90	87.80	55.20
YOLOv13n	2.45	5.8	5.0	88.43	84.52	91.10	54.93
ME-YOLO	2.75	6.5	5.7	94.8	89.7	88.5	-
Ours	1.55	3.8	3.3	91.70	87.80	92.40	60.00

Note: The symbol “-” indicates that the corresponding metric was not reported in the original reference paper.

**Table 9:** Comparison of experimental results on NEU-DET generalization performance.

Model	Precision/%	Recall/%	mAP@0.5/%	Params/106	GFLOPs
YOLO11n	69.2	69.3	76.0	2.73	5.9
Ours	71.9	70.2	76.4	1.57	3.6

## Data Availability

Data are contained within the article.

## Abbreviations

The following abbreviations are used in this manuscript:

YOLO	You Only Look Once
POLD-YOLO	PoolingFormer OD-ADown LSCD-Head Focaler-MPDIoU-YOLO
C3K2	Cross-Stage Partial block with a kernel size of 2
C3K2-PFCGLU	C3K2-PoolingFormer and Convolutional Gated Linear Unit
OD-ADown	Omni-Dimensional Adaptive Downsampling
LSCD-Head	Lightweight Shared Convolutional Detection Head
Focaler-MPDIoU	Focaler-Minimum Point Distance based IoU
UAV	Unmanned Aerial Vehicle
P	Precision
R	Recall
mAP	Mean Average Precision
GFLOPs	Giga Floating Point Operations
Params	Parameter Counts
